# Pressureless Immersion of Epoxy Resin-Filled Cracks in Faulted Rock Materials

**DOI:** 10.3390/ma17133111

**Published:** 2024-06-25

**Authors:** Kui Yu, Yong She, Jibing Chen, Xionghui Cai, Yiping Wu

**Affiliations:** 1 School of Mechanical Engineering, Wuhan Polytechnic University, Wuhan 430023, China; 2 School of Materials Science and Technology, Huazhong University of Science and Technology, Wuhan 430074, China

**Keywords:** epoxy resin, faulted rock, penetration experiment, strength test

## Abstract

Epoxy resin, known for its excellent corrosion resistance, water resistance, and high-temperature resistance, is extensively utilized in construction and water-related projects. Within water conservancy projects, natural factors such as water impact and weathering often result in cracks within rock formations. Consequently, the application of epoxy resin materials for repair and reinforcement has emerged as a common solution. This research investigates the impact of five epoxy grouting materials, YDS (100:6.4), RH-1 (6.1:1), PSI (9:1), TK (100:8), and HK-G (5:1), on the repair and reinforcement of faulted rock at the Yebatan Hydropower Station. Penetration experiments were conducted on rock samples, and the strength of the epoxy grout samples was tested under ambient conditions of 20 °C, 15 °C, and 0 °C. The experimental results indicate that all five epoxy grout materials successfully penetrated the faulted rock samples. Among them, the PSI (9:1) epoxy grouting material exhibited the most exceptional reinforcing effect across different temperatures, with grouting samples demonstrating strengths in the range of 20 to 25 MPa. This paper confirms that epoxy resin effectively repairs and reinforces rock structures, thereby enhancing the safety and durability of water conservancy projects.

## 1. Introduction

Epoxy resin (EP) adhesive is a high value-added adhesive, with a large adhesive force, high bond strength, excellent chemical stability, low shrinkage, ease of processing and molding, and eco-friendliness. It exhibits remarkable bonding capabilities with metal, glass, wood, plastics, ceramics, composites, cement, rubber, fabrics, and other polar materials, making it extensively utilized in civil engineering, construction, water conservancy, and various other projects [1,2,3]. In recent years, various types of high-performance EP adhesives have been developed, including high-temperature, high-toughness, moisture-resistant, and room-temperature curing variants [4,5,6]. Epoxy resin grouting material offers distinct advantages over traditional options, boasting a low shrinkage rate, the ability to fill small cracks and voids, and a strong bonding capability between different materials. Additionally, its curing time is controllable, enhancing its engineering applicability [7,8,9]. As an emerging chemical grouting material, epoxy resin assumes an increasingly vital role in the grouting field. Its primary components include an epoxy resin, curing agent, diluent, toughening agent, filler, aggregate, and other additives. Only by selecting the appropriate components and proportions can the ideal epoxy grouting material be formulated [10].

When the epoxy resin is utilized in wet or underwater environments, a weak interfacial layer often forms between the adhesive and the adherent, which is not easy to cure. Additionally, the high viscosity of epoxy resin at room temperature makes it difficult to meet the requirements of fluidity and permeability during the actual application process [11,12]. Currently, numerous experimental studies have been conducted both domestically and internationally on the dosage of components in epoxy grouting materials, as well as their construction performance, adhesive properties, water resistance, and other factors. Wang et al. [13] modified epoxy resin by incorporating polydimethylsiloxane (PDMS) and a diluent, and investigated the mechanical properties of the resulting modified materials. It was demonstrated that the strength and impact resistance of the silicone-based modified epoxy grouting materials were significantly improved. Moreover, the flexibility and bonding properties of the modified materials surpassed those of the unmodified ones, which enhanced the environmental adaptability of the materials. Wang et al. [14] combined acetone with either 1,6-hexanediol diglycidylether (RDDF) or trimethylolpropane glycidylether (RDTF) to create two diluents for the formulation of ultra-low viscosity epoxy grouting materials. The experimental results indicated that the modification of epoxy groups accelerated the reaction with curing agents and ether groups, altering the brittleness of epoxy materials. Additionally, the addition of composite diluents significantly reduced the viscosity of epoxy grouting materials, facilitating the repair of microcracks in buildings. Wang et al. [15] optimized the preparation process of silicone-modified low-viscosity epoxy grouting materials and investigated the evolution of their mechanical properties under conditions of high-temperature impact, temperature cycling, and freeze–thaw cycling. The experimental results indicated that the physical and mechanical properties of the grouting materials were optimal when prepared at a reaction temperature of 100 °C, a reaction time of 3 h, and a dichlorodimethylsilane (DDMS) content of 11%. After 3 days of curing, the shear strength and bond strength of the material reached over 90% at room temperature and between 70% to 90% at high temperatures. Wang et al. [16] incorporated water-soluble epoxy resin into cement-based grout and investigated the rheological and mechanical properties of the composite grouting materials. The results showed that the fluidity of the grouting material increased and then decreased with the addition of epoxy resin. Moreover, the uniaxial compressive strength and split tensile strength of the modified material experienced significant improvements, and the bonding ability of the material was enhanced. Zhang et al. [17] utilized flexible hexamethylene diisocyanate (HDI) to synthesize a novel high-toughness epoxy resin through copolymerization. The experiment demonstrated significant enhancements in the mechanical properties of the modified epoxy resin material, with an elongation at break reaching as high as 124%. In addition, the specimens exhibited thermal stability up to 258 °C and excellent corrosion resistance; they remained stable in H_2_SO_4_, NaOH, and 10 wt% NaCl solutions for 100 h. Liu et al. [18] synthesized nano-silica (NS)-modified waterborne epoxy resin (WEP) through in situ polymerization and analyzed the mechanical properties of the grouting material. The findings indicated that increasing the NS concentration significantly reduces the solidification time of the grouting material while enhancing the compressive strength, stiffness, and toughness of the specimens. Notably, when the NS content in WEP reaches 4%, the stress–strain curve exhibits a distinct stress step, signifying the optimal mechanical properties of the specimen. Wang et al. [19] investigated the mechanical properties of grouting materials by diluting epoxy resin using a low-viscosity reactive diluent and anhydrous ethanol. Experimental results revealed a compressive strength of 40.6 MPa and a tensile strength of 12.6 MPa for the thinned epoxy material post-solidification, and infrared spectra analysis indicated an enhancement in the toughness of the epoxy material due to the inclusion of the diluent. Zhang et al. [20] examined four common grouting materials—ordinary silicate cement, epoxy resin, ultrafine cement, and polyurethane—and conducted comprehensive analyses, considering factors such as grouting pressure and concrete crack aperture. They investigated the anti-seepage performance of each material on concrete cracks. The experimental findings reveal that epoxy resin demonstrates the most superior anti-seepage performance, establishing it as the optimal grouting material. Furthermore, the article delves into the differences in the water plugging efficacy among the four materials, exploring the microstructure of grouted concrete cracks as a contributing factor. The penetration performance of epoxy resin grouting material may deteriorate due to prolonged placement time. Su et al. [21] investigated this phenomenon and analyzed the impact of time on the viscosity and affinity of epoxy resin grouting material. They examined the properties such as contact angle and viscosity of CW epoxy resin grouting material with different mass ratios of A:B = 5:1 and 6:1. Furthermore, the researchers established a mathematical model to describe the change in viscosity and affinity of epoxy resin grout over time, providing a detailed analysis of the model. Experimental results revealed that the surface tension of both types of epoxy grouting materials increased over time and eventually reached equilibrium. Moreover, as the experimental duration extended, the contact angle of both CW epoxy grouting materials increased, while the affinity decreased. Consequently, the permeability of the epoxy resin grouting material was weakened.

In this paper, anti-permeability reinforcement experiments were conducted on the faulted rock formations within the vicinity of the Yebatan Hydropower Station These geological formations are characterized by sandy granular material exhibiting low hardness and inadequate impermeability. Traditional cement grouting methods have proved ineffective in fortifying these structures against seepage, necessitating recourse solely to chemical grouting techniques. Due to the geographical proximity of the chosen faulted rock samples to the water layer’s surface, there exists no requirement for applying external pressure during experimentation [22].

## 2. Experiments

**Figure 1:**
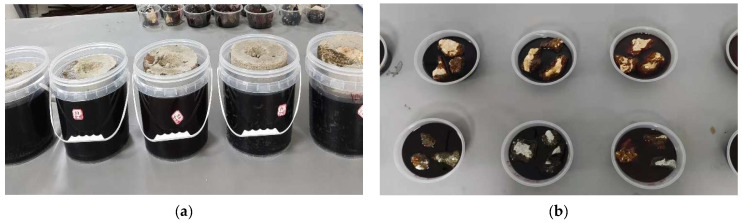
(**a**) rock penetration test immersion; (**b**) rock strength test immersion.

In this experiment, two fault rock bodies, namely F2 (the density is 3.3 g/cm^3^) and F3 (the density is 2.7 g/cm^3^), located at different sites within the Yebatan Hydropower Station, were chosen for conducting permeability and strength experiments, as illustrated in Figure 1. Five commercially available epoxy resin grouting materials were selected for evaluation, as specified in Table 1.

The epoxy grouting material is required to penetrate into the rock body to enhance its hardness, thus necessitating a penetration depth test [23,24,25,26]. Due to the loose nature of the rock bodies in faults F2 and F3, they tend to disperse when soaked in water. Therefore, before experimentation, they were encapsulated with cement slurry and subsequently placed into containers. The epoxy grouting material was prepared according to the proportions outlined in Table 2. Component A was composed of epoxy resin and diluent (alkyl glycidyl ether, HK-66) in a proportion of 9:1. Component B was generally a curing system. The curing agent in the curing system was ethylenediamine, and the mixing procedures are as follows: (1) Component A is prepared in the desired proportion and evenly mixed for 1 h; (2) Component A is mixed at room temperature according to the ratio of A: B in Table 2; (3) the prepared epoxy grouting material is poured into a container equipped with fault rock mass and the time is recorded; (4) epoxy resin is cured for 28 days at room temperature. The epoxy resin is infiltrated in the rock samples to cure at room temperature, after which the cured material is sectioned to measure the penetration depth of the samples.

**Figure 2:**
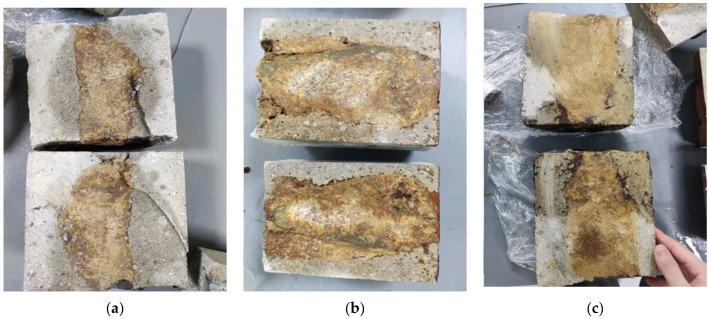
F3 epoxy cured casting body sections: (**a**) RH (6:1) epoxy casting body section; (**b**) PSI (9:1) epoxy casting body section; (**c**) YDS (100:6.4) epoxy casting body section.

The fault rock F3 exhibits a highly porous and easily penetrable nature. Therefore, three epoxy resin materials with relatively high penetrability, namely RH (6:1), PSI (9:1), and YDS (100:6.4), were selected as representatives for the penetration depth experiment. As depicted in Figure 2, the deep yellow regions represent areas penetrated by the epoxy grout. It is evident that the cut surfaces of the three pours darken uniformly after curing, indicating complete penetration. This underscores that the penetration rate of the three epoxy grouting materials with relatively high penetrability into the rock body of F3 reaches 100%.

**Figure 3:**
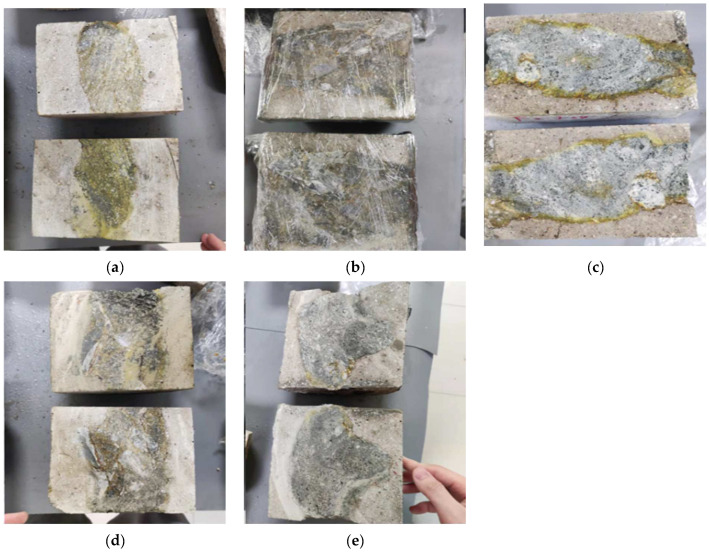
F2 epoxy cured casting body sections: (**a**) Pascal (9:1) epoxy casting body section; (**b**) YDS (100:6.4) epoxy casting body section; (**c**) HK-G (9:1) epoxy casting body section; (**d**) TK (100:8) epoxy casting body section; (**e**) RH (6:1) epoxy casting body section.

Compared with sample F3, rock sample F2 has more internal cracks and a harder texture, necessitating penetration depth experiments with five epoxy grouting materials. As illustrated in Figure 3, PSI epoxy grout (Figure 3a), YDS epoxy grout (Figure 3b), and TK epoxy grout (Figure 3d) exhibit yellowish traces within the rock fissures, indicating complete penetration of these three epoxy resin formulations into the rock body of the F2 fault. Figure 3c shows the cross-section of the casting body following immersion in HK-G (6:1) epoxy grout. Most of this epoxy grout material only penetrated around the F2 rock body, and a small piece was penetrated in the middle; this limited penetration can be attributed to the higher density and harder texture present in the middle of this rock sample, coupled with the high initial viscosity of the HK-G (6:1) epoxy grout material, resulting in unsatisfactory penetration. Figure 3e illustrates a cross-section of the casting body after immersion in RH (6:1) epoxy grout. The epoxy grout only penetrated a small portion at the front of the rock body. This limited penetration was likely due to the protective cement grouting applied to the F2 rock body, preventing full contact with the epoxy grout during the cement grouting process, thus impeding the epoxy grout’s penetration.

To determine the epoxy grouting material with optimal strength enhancement for the faulted rock body of the Yebatan Hydropower Station, it is imperative to conduct strength testing experiments on the samples [27,28,29]. The effect of HK-G (9:1) epoxy grouting material on F2 rock penetration was not obvious in the rock penetration experiments, so the viscosity of HK-G epoxy was reduced in the experiments. The epoxy grouting materials were formulated according to the proportions outlined in Table 3, for conducting pressure-free immersion tests on the fault rock body. Given that the rock bodies of F2 and F3 faults are subject to environmental influences such as low temperatures, water scouring, and weathering, the epoxy resin casting bodies underwent a 20-day curing process at both 0 °C and ambient temperature (20 °C) before conducting strength tests to assess and screen the epoxy grouting materials.

### 2.1. Strength Test Analysis of Epoxy Resin Casting Body at 0 °C

**Figure 4:**
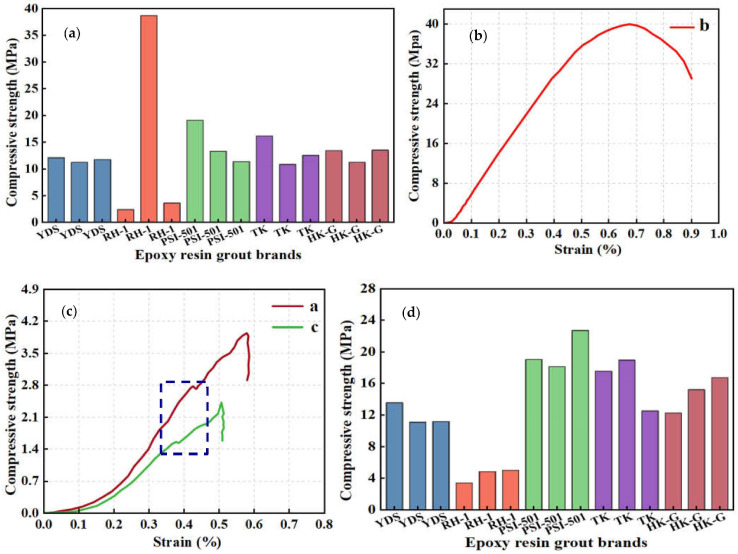
(**a**) Strength test values of F2 rock mass after curing at 0 °C; (**b**) Stress–strain curves of RH (6:1) cast bodies of group b; (**c**) Stress–strain curves of RH (6:1) cast bodies of groups a and c; (**d**) Strength test values of F3 rock mass after curing at 0 °C.

As depicted in Table 4 and Figure 4a, the compressive strength of the F2 casting body following immersion curing with YDS (100:6.4) epoxy grout was approximately 11 MPa; the compressive strength of the TK (100:8) epoxy casting body measured around 13 MPa. Meanwhile, the compressive strength of the F2 casting body after immersion curing for PSI (9:1) and HK-G (5:1) were around 14 MPa and 13 MPa, respectively.

The casting body treated with RH (6:1) epoxy grout dispersed upon cutting, indicating that despite the epoxy resin material achieving 100% penetration into the F2 rock, it exhibited limited curing efficacy at low temperatures, and it primarily transformed the F2 rock body from an initial state of gravelly granular composition, containing sand and gravel, to a solidified state resistant to dispersion. Notably, the strength of the second experimental (b) epoxy casting of RH (6:1) is significantly higher than that of the first (a) and third (c) epoxy castings. Consequently, stress–strain analyses were conducted, as illustrated in Figure 4b,c.

The stress–strain curve of the epoxidized rock sample of the second experiment (b) exhibits a long deformation stage and a large slope, and reaches the crushing point after the deformation stage, which is similar to the stress–strain diagrams of hard rock, indicating that this rock sample possesses high hardness before immersion. Conversely, the stress–strain curves of the epoxy resin rock samples from the first (a) and third (c) experiments show a nonlinear compression–density phase, an elastic phase, and a brittle damage phase, akin to stress–strain diagrams observed in rocks containing sandy particles. Notably, a partially decreasing and non-smooth zone appears in the linear section of the curve (as indicated in the figure). The analysis suggests that this is due to the unsatisfactory curing effect of the rock samples and the closure of the rock cracks during the experiment.

Upon reviewing Table 4 and Figure 4d, under a temperature of 0 °C, it was observed that the strength of F3 rock samples after immersion curing with YDS (100:6.4) epoxy grout reached approximately 12 MPa; the strength of F3 rock samples following immersion curing with PSI (6:1) epoxy grout was notably higher, at around 20 MPa, demonstrating exceptional performance. Additionally, the strength of TK (100:8) epoxy rock samples measured approximately 15 MPa, while the amount for HK-G (5:1) was around 16 MPa after immersion curing. Rock dispersion occurred during cutting of F3 rock samples after immersion curing of RH (6:1) epoxy grout material; this phenomenon can be attributed to the transformation of the rock body by the epoxy grouting material, converting it from an initial state containing gravelly granular stone to a solidified state that is less prone to scattering. The efficacy of low-temperature curing remains unsatisfactory.

### 2.2. Strength Test Analysis of Epoxy Resin Casting Body at 20 °C

**Figure 5:**
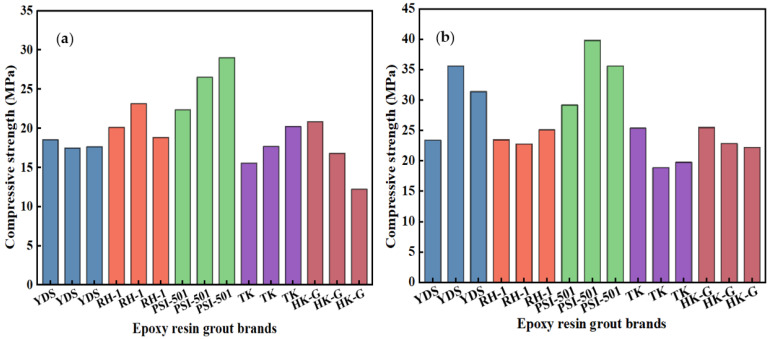
(**a**) Strength test values of F2 rock mass after curing at 20 °C; (**b**) Strength test values of F3 rock mass after curing at 20 °C.

The F2 fault rock casting experiments conducted at 20 °C are shown in Table 5 and Figure 5a. The average value of three measurements of compressive strength of the rock samples following immersion curing with YDS (100:6.4), RH-1 (6:1), PSI (9:1), TK (100:8), and HK-G (6:1) epoxy grout was approximately 18 MPa, 21 MPa, 26 MPa, 18 MPa, and 16 MPa, respectively. It is noteworthy that PSI (9:1) epoxy resin exhibited significant strength enhancement for F2 rock samples.

The experimental data for F3 fault rock within the 20 °C environment are presented in Table 5 and Figure 5b. The average value of three measurements of compressive strength of the rock samples following soaking and curing with YDS (100:6.4) epoxy grout was approximately 30 MPa, while that after treatment with PSI (9:1) epoxy grout it reached around 35 MPa. Furthermore, the compressive strength of F3 rock samples after immersion curing with TK epoxy grout averaged approximately 20 MPa, the compressive strength of F3 rock samples after immersion curing with HK-G (5:1) epoxy grout was approximately 23 MPa, and a comparable strength level of around 23 MPa was observed after immersion curing with RH (6:1) epoxy grout. It is noteworthy that F3 rock samples exhibited better properties than F2 rock samples after curing.

Based on the aforementioned experimental data, it can be concluded that the PSI (9:1) epoxy grout exhibits the most effective curing performance on the rock samples from F2 and F3 faults at 20 °C. This is due to two reasons. On the one hand, the diluent in PSI (9:1) is alkylene glycidyl ether (HK-66), which contains epoxy groups, which can participate in the curing reaction and forms a three-dimensional cross-linked ring structure, so it has a better enhancement effect on the mechanical properties of fault rock mass. On the other hand, upon comparison of the experimental results from both samples, it is evident that the epoxy grout utilized for strengthening the F3 rock body demonstrates significantly higher strength values compared to those of the F2 rock body. This is because the relatively looser nature of the F3 rock body facilitates easier penetration of the epoxy grouting material, thereby resulting in superior curing effects.

Additionally, in conjunction with strength test experiments conducted on epoxy resin rock samples at temperatures of 0 °C and 20 °C, it was observed that the RH (6:1) epoxy grouting material is suboptimal for anti-seepage reinforcement of the fault samples F2 and F3 from the Yebatan Hydropower Station under low-temperature conditions. By reducing the viscosity of HK-G epoxy grouting material, the penetration capability of the grouting material was notably enhanced, consequently leading to increased strength of the rock samples from F2 and F3. Notably, among the tested materials, the PSI (6:1) epoxy grouting material exhibited the most remarkable enhancement in sample strength at both 0 °C and 20 °C.

### 2.3. Strength Test Analysis of Epoxy Resin Casting Body at 15 °C

**Figure 6:**
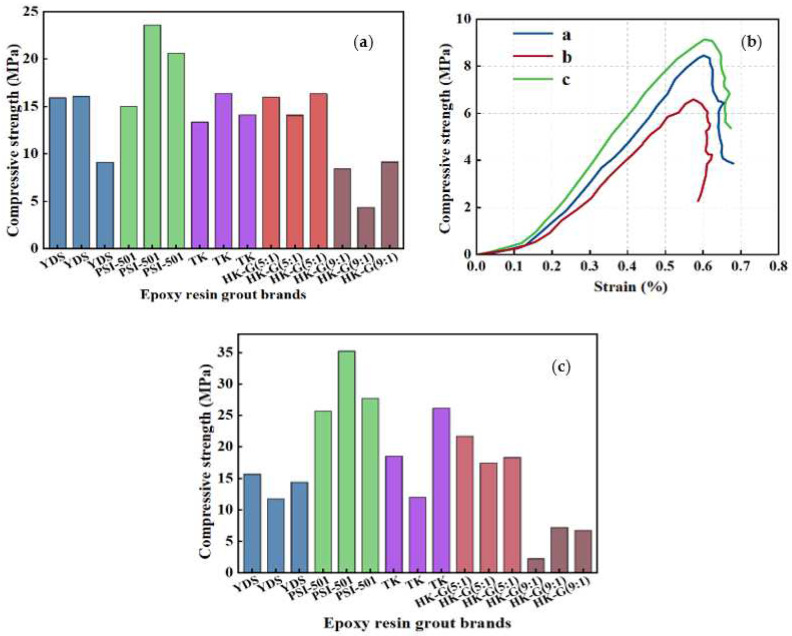
(**a**) Strength test values of F2 rock mass after curing at 15 °C; (**b**) Stress–strain curve of HK-G (5:1) casting body; (**c**) Strength test values of F3 rock mass after curing at 15 °C.

In order to verify that the concentration of HK-G epoxy grouting material affected the effect of penetrating the rock mass, two epoxy grouting materials with different concentrations, HK-G (5:1) and HK-G (9:1), were used for the strength tests, as shown in Table 6 and Figure 6a.

Analyzing the experimental data of two viscosities of HK-G epoxy grout at 15 °C, it can be found that the strength of HK-G (5:1) epoxy grout after immersion curing was approximately 15 MPa for F2 rock samples and around 19 MPa for F3 rock samples. The strengths of the F2 rock samples after immersion curing with HK-G (9:1) epoxy grout were approximately 8 MPa, while those of the F3 rock samples were around 7 MPa. These results indicate that the curing efficacy of HK-G (5:1) is superior to that of HK-G (9:1).

During the experimentation process, apart from observing the dispersion phenomenon while cutting rock samples treated with HK-G (9:1) epoxy resin grouting material, it was observed that the epoxy resin did not penetrate as well as it should have and that the sand particles in the rock failed to coagulate into a single unit. The stress–strain curves of the F2 rock samples from the first experiment (a), the second experiment (b) and the third experiment (c) were analyzed as shown in Figure 6b. The curves of the three experimental samples were the stress–strain diagrams of the rock body containing sandy granular particles, akin to those observed in the RH (6:1) rock samples in Figure 4c. This observation further confirms the inadequate curing efficacy of RH (6:1) in low-temperature environments, wherein it only converts sandy gravelly rock bodies into solidified forms less prone to dispersion.

In the experimental environment of 15 °C, the PSI (9:1) epoxy grout cured the rock samples best among the five epoxy resin materials, with the strength of the cured F2 rock samples measuring around 20 MPa, and approximately 30 MPa for the F3 rock samples, indicating excellent performance. In addition, the curing time of epoxy resin at 15 °C was significantly longer than that at 20 °C. However, despite the prolonged curing time, the increase in strength of epoxy resin rock samples did not vary significantly. The reason is that the epoxy resin grout reaches a saturation point in terms of rock body penetration, whereby further extension of the curing time has minimal impact on the strength of the rock samples.

## 3. Conclusions

In this paper, five different epoxy resin grouting materials, YDS (100:6.4), RH-1 (6.1:1), PSI (9:1), TK (100:8), and HK-G (5:1), were used to conduct penetration and strength enhancement tests on the faulted rock of the Yebatan Hydropower Station. The experimental results showed that:

1. As the temperature decreases, the curing time of the same proportion of epoxy grouting material increases. However, once the curing time reaches a certain threshold, the effect on the strength of the epoxy rock samples becomes less pronounced.
2. Among different temperature conditions, the PSI (9:1) epoxy grouting material demonstrates superior enhancement of the F2 and F3 fault rock samples’ strength, yielding the best overall performance. Conversely, the RH-1 (6.1:1) epoxy grouting material exhibits unsatisfactory curing effects on the F2 and F3 samples under low-temperature conditions.
3. Reducing the viscosity of the epoxy grouting material enhances the penetration effect on the samples. Compared to the HK-G (9:1) epoxy grouting material, the HK-G (5:1) epoxy grouting material leads to a strength enhancement of approximately 10 MPa in cured samples in the F3 fault rock.

In summary, for enhancing the strength of fault rock bodies at Yebatan Hydropower Station, the PSI epoxy grouting material is preferred, with adjustments needed in curing time when applying epoxy grouting material under low temperatures.

## Figures and Tables

**Table 1:** Epoxy resin grout brands and R&D Units.

Epoxy Resin Grout Brands	R&D Units
YDS	Guangzhou Institute of Chemistry, Chinese Academy of Sciences
RH-1	Ripar Technology Group
PSI-501	Pascal Limited
TK	Guangdong Tieke Grouting Technology Co.
HK-G	Hangzhou Guodian Hydropower Engineering Co.

**Table 2:** Proportions of epoxy grouting materials.

Epoxy Resin Grout Brands	Ratio (A:B)	Immersion Rock Sample
YDS	100:6.4	F2, F3
RH-1	6:1	F2, F3
PSI-501	9:1	F2, F3
TK	100:8	F2, F3
HK-G	6:1	F2, F3

**Table 3:** Proportions of epoxy grouting materials at 0 °C and 20 °C.

Epoxy Resin Grout Brands	Ratio (A:B)	Immersion Rock Sample
YDS	100:6.4	F2, F3
RH-1	6:1	F2, F3
PSI-501	9:1	F2, F3
TK	100:8	F2, F3
HK-G	5:1	F2, F3

**Table 4:** Strength test data after curing of the rock mass.

Brands	Ratio (A:B)	Gel Time/h	Curing Temperature	Sample	Compressive Strength /MPa	Maximum Test Force/KN	Sample	Compressive Strength/MPa	Maximum Test Force/KN
					12.13	5.26		13.55	6.04
YDS	100:6.4	680 (28 d)	0 ℃	F2	11.27	4.93	F3	11.09	4.82
					11.78	5.13		11.17	4.88
					2.43	1.24		3.43	1.82
RH -1	6:1	70 (3 d)	0 ℃	F2	38.72	16.03	F3	4.86	2.41
					3.67	1.63		5.03	2.53
				F2	19.14	8.36	F3	19.03	8.28
PSI-501	6:1	450 (19 d)	0 ℃		13.35	5.93		18.13	7.83
					11.4	4.94		22.69	9.62
					16.19	6.73		17.55	7.53
TK	100:8	470 (20 d)	0 ℃	F2	10.89	4.73	F3	18.96	8.22
					12.57	5.58		12.52	5.51
					13.45	5.96		12.25	5.32
HK-G	5:1	600 (25 d)	0 ℃	F2	11.27	4.89	F3	15.21	6.56
					13.56	6.12		16.74	7.06

**Table 5:** Strength test data after curing of the rock mass.

Brands	Ratio (A:B)	Gel Time/h	Curing Temperature	Sample	Compressive Strength /MPa	Maximum Test Force/KN	Sample	Compressive Strength /MPa	Maximum Test Force/KN
					18.55	8.01		23.41	9.91
YDS	100:6.4	240	20 °C	F2	17.5	7.53	F3	35.6	14.95
					17.65	7.61		31.37	12.91
					20.13	8.33		23.45	9.98
RH -1	6:1	24	20 °C	F2	23.14	9.71	F3	22.76	9.63
					18.85	8.12		25.10	10.79
				F2	22.36	9.57	F3	29.18	12.36
PSI-501	6:1	84	20 °C		26.52	11.03		39.8	16.23
					29.03	11.94		35.59	14.55
					15.56	6.58		25.38	10.58
TK	100:8	85	20 °C	F2	17.69	7.62	F3	18.88	8.18
					20.23	8.44		19.76	8.38
					20.84	8.53		25.48	10.61
HK-G	5:1	30	20 °C	F2	16.8	7.04	F3	22.85	9.84
					12.22	5.23		22.18	9.43

**Table 6:** Strength test data after curing of the rock mass.

Brand	Ratio (A:B)	Gel Time/h	Curing Temperature	Sample	Compressive Strength /MPa	Maximum Test Force/KN	Sample	Compressive Strength /MPa	Maximum Test Force/KN
					15.90	6.63		15.66	6.59
YDS	100:6.4	300 (13 d)	15 °C	F2	16.06	6.78	F3	11.75	5.12
					9.11	4.31		14.36	6.14
					15.01	6.53		25.67	10.82
RH -1	6:1	82	15 °C	F2	23.57	10.01	F3	35.24	14.35
					20.6	9.09		27.73	11.68
				F2	13.34	5.93	F3	18.5	7.96
PSI-501	6:1	140 (5 d)	15 °C		16.35	6.98		11.98	5.16
					14.07	6.22		26.16	10.84
					15.98	6.67		21.69	9.19
HK-G	5:1	140 (5 d)	15 °C	F2	14.06	6.22	F3	17.41	7.42
					16.32	6.93		18.31	7.84
					8.45	3.76		8.33	3.26
HK-G	9:1	160 (7 d)	15 °C	F2	6.59	2.61	F3	7.21	3.23
					9.13	4.09		6.74	2.77

## Data Availability

The raw data supporting the conclusions of this article will be made available by the authors on request.
